# Collectanea Medica

**Published:** 1813-05

**Authors:** 


### COLLECTANEA MEDICA,

CONSISTING OF

ANECDOTES, FACTS, EXTRACTS, ILLUSTRATIONS, QUERIES, SUGGESTIONS, &c.

RELATING TO THE

*History or the Art of Medicine, and the Auxiliary Sciences.*

---

### *A Biographical Account of the Honorable Henry Cavendish.* *By* THOMAS THOMSON, M.D. F.R.S.

THE Hon. Henry Cavendish was born in London, on the 10th of October, 1731. His father was Lord Charles Cavendish, a cadet of the family of Devonshire, one of the oldest houses in England. During his father's lifetime he was kept rather in narrow circumstances. His father allowed him an annuity of 500*l.*, and fitted up his stables for his accommodation, where he lived for many years. It was during this period that he acquired those habits of economy, and those singular oddities of character, which he exhibited ever afterwards in so striking a manner. At his father's death he was left a very considerable fortune; and an aunt, who died at a later period, bequeathed him a very handsome addition. In consequence of the habits of economy which he had acquired, it was not in his power to spend the greater part of his his annual income. This occasioned a yearly increase to his capital, till at last it accumulated so much, without any care on his part, that at the period of his death he left behind him nearly 1,300,000*l.*, and was the greatest proprietor in the Bank of England. On one occasion, his money in the hands of his bankers accumulated to the amount of 70,000*l.* These gentlemen, thinking it improper to keep so large a sum in their hands, sent one of the partners to wait upon him, in order to learn how he wanted it disposed of. This gentleman was admitted, and, after employing the necessary precautions to a man of Mr. Cavendish's peculiar disposition, stated the circumstance, and begged to know whether it would not be proper to lay out the money. Mr. Cavendish drily answered, " You may lay it out if you please," and left the room.

Mr. Cavendish hardly ever went into any other society than that of his scientific friends. He never was absent from the weekly dinner of the Royal Society Club, at the Crown and Anchor Tavern. At these dinners, when he happened to be seated near those that he liked, he often conversed a great deal; though at other times he was very silent. He was, likewise, a constant attendant at Sir Joseph Banks's Sunday evening meetings. He had a house in London, which he only visited once or twice a-week at stated times, and without ever speaking to the servants. It contained an excellent library, to which he gave all literary men the freest and most unrestrained access. But he lived in a house on Clapham Common, where he scarcely ever received any visitors. His relation, Lord George Cavendish, to whom he left by will the greatest part of his fortune, visited him only once a-year; and the visit hardly ever exceeded ten or twelve minutes.

He was shy and bashful, to a degree bordering upon disease. He could not bear any person to be introduced to him, or to be pointed out, in any way, as a remarkable man. One Sunday evening, he was standing at Sir Joseph Banks's, in a crowded room, conversing with Mr. Hatchett, when Dr. Ingenhousz, who had a good deal of pomposity of manner, came up, with an Austrian gentleman in his hand, and introduced him formally to Mr. Cavendish. He mentioned the titles and qualifications of his friend at great length, and said that he had been peculiarly anxious to be introduced to a philosopher so profound, and so universally known and celebrated, as Mr. Cavendish. As soon as Dr. Ingenhousz had finished, the Austrian gentleman began, and assured Mr. Cavendish, that his principal reason for coming to London was to see and converse with one of the greatest ornaments ments of the age, and one of the most illustrious philosophers that ever existed. To all these high-flown speeches Mr. Cavendish answered not a word; but stood with his eyes cast down, quite abashed and confounded. At last, spying an opening in the crowd, he darted through it with all the speed he was master of; nor did he stop till he reached his carriage, which drove him directly home.

Mr. Cavendish died on February the 4th, 1810, aged 78 years, four months, and six days. His appearance did not much prepossess strangers in his favor. He was somewhat above the middle size, his body rather thick, and his neck rather short. He stuttered a little in his speech, which gave him an air of awkwardness. His countenance was not strongly marked, so as to indicate the profound abilities which he possessed. This was probably owing to the total absence of all the violent passions. His education seems to have been very complete. He was an excellent mathematician, a profound electrician, and a most acute and ingenious chemist. He never ventured to give an opinion upon any subject, unless he had studied it to the bottom. He appeared before the world first as a chemist, and afterwards as an electrician. The whole of his literary labors consist of 17 papers, published in the Philosophical Transactions, and occupying each only a few pages; but full of the most important discoveries, and the most profound investigations. Of these papers there are ten which treat of chemical subjects, two treat of electricity, two of meteorology, and three are connected with astronomy. Let us take a view of these papers, in the order in which we have mentioned them.

I. *Chemical Papers.*—Mr. Cavendish's first paper was published in the year 1766, when he was 35 years of age. It was entitled, *Experiments on Factitious Air*, and constituted a most important step in the science of chemistry. Dr. Hales had demonstrated that *air* is given out by a vast number of bodies in peculiar circumstances; but he never suspected that any of the airs which he obtained differed from *common air*. Indeed, common air had always been considered as an elementary substance, to which every elastic fluid was referred. Dr. Black had demonstrated that calcareous spar and the mild alkalies differed from quick-lime and the caustic alkalies, in containing a quantity of air, chemically combined with the lime and the alkaline bodies. He called this air *fixed air*; and, though he had not examined its properties, there was reason to conclude, from the observations which he made, that *fixed air* was not of the same nature with common air. Mr. Cavendish, in this paper, demonstrates that there are two species of air quite different in their properties

 from from common air. These two are *inflammable air* and *fixed air*. He mentions, likewise, a third species of air; namely, that given out when metals are dissolved in nitrous acid. It differed, as he showed, from the other species, though he did not examine its properties in detail.

The inflammable air (now known by the name of *hydrogen gas*) was obtained by dissolving iron, zinc, or tin, in diluted sulphuric or muriatic acids. Iron yielded about $\frac{1}{22}$ part of its weight of inflammable air, zinc about $\frac{1}{23}$ or $\frac{1}{24}$th of its weight, and tin about $\frac{1}{44}$th of its weight. The properties of the air discharged were the same, whichever of the three metals were used; and whether they were dissolved in sulphuric or muriatic acids. When the sulphuric acid was concentrated, iron and zinc dissolved in it with difficulty, and only by the assistance of heat. The air given out was not inflammable, but consisted of sulphuric acid. These facts induced Mr. Cavendish to conclude, that the inflammable air evolved in the first case consisted of the unaltered phlogiston of the metals, the sulphurous acid of the same phlogiston united to a portion of the acid, which deprived it of its inflammability. He found the specific gravity of his inflammable air about 11 times less than that of common air. This determination is somewhat less than the truth; but the error is probably owing chiefly to the quantity of water held in solution by the air, and which Mr. Cavendish showed amounted to $\frac{1}{9}$th of the weight of the air. He tried the combustibility of the inflammable air when mixed with various proportions of common air, and found that it exploded with the greatest violence when mixed with rather more than its bulk of common air.

Copper, he found, when dissolved in muriatic acid by the assistance of heat, yielded no inflammable air; but an air which lost its elasticity when it came in contact with water. This air, the nature of which Mr. Cavendish did not examine, was muriatic acid gas, the properties of which were soon after investigated by Dr. Priestley.

The fixed air (now known by the name of *carbonic acid gas*), on which Mr. Cavendish made his experiments, was obtained by dissolving marble in muriatic acid. He found that it might be kept over mercury for any length of time, without undergoing any alteration; that it was gradually absorbed by cold water; and that 100 measures of water of the temperature of 55° absorbed 103·8 measures of fixed air. The whole of the air thus absorbed was separated again by exposing the water to a boiling heat, or by leaving it for some time in an open vessel. Alcohol (the specific gravity of which is not mentioned) dissolved $2\frac{1}{4}$ times its bulk of this air, air, and olive oil about $\frac{1}{3}$d of its bulk. The specific gravity of fixed air he found 1·57, that of common air being 1·. Fixed air is incapable of suppórting combustion; and common air, when mixed with it, supports combustion a much shorter time than when pure. A small wax taper burnt 80″, in a receiver which held 180 ounce measures, when filled with common air only. The same candle burnt 51″ in the same receiver, when filled with a mixture of 1 part of fixed air and 19 of common air. When the fixed air was $\frac{3}{40}$ of the whole mixture, the candle burnt 23″. When the fixed air was $\frac{1}{10}$th of the whole, it burnt 11″. When the fixed air was $\frac{6}{55}$ or $\frac{1}{9\cdot16}$ of the whole mixture, the candle went out immediately.

Mr. Cavendish conceived that the nature of the fixed air given out by marble differed somewhat; or that the elastic fluid emitted consisted of two airs, one more absorbable by water than the other. He drew his conclusion from the circumstance, that after a solution of potash had been exposed to a quantity of fixed air for some time, it ceased to absorb any more; yet, if the residual portion of air were thrown away, and new fixed air substituted in its place, it began to absorb again. But Mr. Dalton long after explained this seeming anomaly in a satisfactory manner, by showing that the absorbability of fixed air by water is proportional to its purity; and that when mixed with a great quantity of common air, or any other gas not soluble in water, it ceases to be sensibly absorbed.

Mr. Cavendish ascertained the quantity of fixed air contained in marble, carbonate of ammonia, common pearl-ashes, and carbonate of potash. But, notwithstanding the great precision with which the experiments were made, these determinations are of comparatively little value; because the proper precautions could not, in that infant state of chemical science, be taken, to have these salts in a state of purity. The following were the results obtained by Mr. Cavendish.

| | | |
|---|---|---|
| 1000 grains of marble contained | 408 grains of fixed air. |
| 1000 -------- carb. of ammonia | 533 |
| 1000 -------- pearl-ashes ----- | 284 |
| 1000 -------- carb. of potash -- | 423 |

Carbonate of potash was first obtained in the state of crystals by Dr. Black. Mr. Cavendish formed it by making a solution of pearl-ashes absorb fixed air till it deposited crystals. He examined the properties of these crystals. They were not altered by exposure to the air, did not deliquesce, and were soluble in about 4 times their weight of cold water.

Dr. M'Bride had already ascertained that vegetable and animal substances yield fixed air by putrefaction and fermentation. Mr. Cavendish found by experiment that sugar, when dissolved in water, and fermented, gives out $\frac{57}{100}$ of its weight of fixed air, possessing exactly the properties of fixed air from marble. During the fermentation no air was absorbed, nor was any change produced upon the common air upon the surface of the fermenting liquor. Apple-juice fermented much faster than sugar; but the phenomena were the same, and the fixed air emitted amounted to $\frac{381}{1000}$ of the weight of the solid extract of apples. Gravy and raw meat yield inflammable air during their putrefaction; the former in much greater quantity than the latter. This air, as far as Mr. Cavendish's experiments went, is the same as the inflammable gas from zinc; but its specific gravity he found a little higher.

Mineral waters have at all times attracted the attention of the medical faculty, in consequence of the peculiar properties which they possess, and the medicinal virtues which they are supposed to exhibit. No sooner had the science of chemistry made any progress, than attempts were made by means of it to ascertain to what the peculiar properties of these waters were owing, and to determine the constituents of which they were respectively composed. Some faint attempts towards the analysis of these waters were made by Boyle. Du Clos attempted to make an experimental analysis of the mineral waters in France; and Hierne published a set of experiments on the mineral waters of Sweden. Though these investigations were rude and inaccurate, they led to the knowledge of several facts respecting mineral waters, which chemists were unable to explain. One of these, and not the least puzzling, was the existence of a considerable quantity of calcareous earth in some mineral waters, which was precipitated by boiling the water. Nobody could conceive by what means this insoluble substance (carbonate of lime) was held in solution; nor why it was thrown down on exposing the water to a boiling heat. It was to determine this point that Mr. Cavendish made his experiments on Rathbone-place water, which were published in the year 1767 (Phil. Trans. vol. lvii. p. 92); and which may be considered as the first analysis of a mineral water, possessed of tolerable accuracy, ever published. All preceding investigations of this kind, when compared with those of Mr. Cavendish, vanish into nothing. Rathbone-place water was at that time raised by a pump, and supplied the streets of London and its neighbourhood with water. Mr. Cavendish found that when boiled it deposited a quantity of earthy matter, matter, consisting chiefly of lime, but containing also a little magnesia. These, he showed, were held in solution by fixed air; and he proved experimentally that this gas has the property of holding lime and magnesia in solution, when an excess of it is present. Besides these earthy carbonates, Rathbone-place water contained a little volatile alkali, some sulphate of lime, some common salt, and a little sulphate of magnesia. Mr. Cavendish examined, likewise, some other pump-water of London; and showed that they contained lime held in solution by carbonic acid.

Dr. Priestley, at a pretty early period of his chemical career, discovered, that when nitrous gas is mixed with common air over water, a diminution of bulk takes place; that there is a still greater diminution of bulk when oxygen gas is employed instead of common air; and that the diminution is proportional to the quantity of oxygen gas present in the gas mixed with the nitrous gas. This discovery induced him to employ nitrous gas as a test of the quantity of oxygen present in common air. And various instruments were contrived to facilitate the mixture of the gases, and the measurement of the condensation. As the goodness of air, or its fitness to support combustion, and maintain animal life, was conceived to depend upon the proportion of oxygen gas which it contained, these instruments were distinguished by the name of eudiometers. The best of them was contrived by the Abbé Fontana, and is usually distinguished by the name of the *Eudiometer of Fontana*. Philosophers, in examining air by means of this instrument, at various seasons, and in various places, had found considerable differences in the diminution of bulks. Hence they inferred that the proportion of oxygen varied; and to this variation they ascribed the healthiness or noxiousness of particular places. Mr. Cavendish examined this important point with his usual patient industry and acute discernment. He ascertained that the apparent variations were owing to inaccuracies in making the experiment; and that, when the requisite precautions were taken, the proportion of oxygen in air was found constant in all places and at all seasons. He determined also, by a correct experiment, that air is a mixture of very nearly 21 parts by bulk of oxygen gas, and 79 parts of azotic gas. (Phil. Trans. 1783, vol. lxxiii. p. 106.)

For many years, it was believed by philosophers that mercury was essentially liquid, and that no degree of cold was capable of congealing it. Professor Braun's accidental discovery, that it is frozen by cold like other liquids, was at first doubted; and, when it was finally established by irrefragable experiments, it was concluded, from the observations of the Petersburgh philosophers, that its freezing point was not less than several hundred degrees below zero. It became an object of great importance to determine the exact point of the congelation of this metal by accurate experiments. This was done at Hudson's Bay by Mr. Hutchins, who followed a set of directions given him by Mr. Cavendish. From these experiments, Mr. Cavendish deduced that the freezing point of mercury is very nearly 39° below zero of Fahrenheit's scale. (Phil. Trans. 1783, vol. lxxiii. p. 303.)

These experiments naturally drew the attention of Mr. Cavendish to the phenomena of freezing, to the action of freezing mixtures, and the congelation of the acids. He employed Mr. M'Nab, who was settled in the neighbourhood of Hudson's Bay, to make requisite experiments. And he published very curious and important papers on these subjects. (Phil. Trans. 1786, vol. lxxvi. p. 241; and 1788, vol. lxxviii. p. 166.) He explained the phenomena of congelation exactly according to the theory of Dr. Black, rejecting only the hypothesis that heat is owing to the presence of a peculiar matter, and thinking it more probable, with Sir Isaac Newton, that it is owing to the rapid internal motion of the particles of the hot body. The latent heat of water he found to be 150°. The observations on the congelation of the nitric and sulphuric acids are highly interesting, but not susceptible of abridgement. He showed that their freezing points varied very considerably, according to the strength of each; and drew up tables indicating the freezing point of acids of various degrees of strength. These papers constitute one of the most interesting, and, perhaps, the best established, part of the theory of heat, as at present taught by chemical philosophers.

But the most splendid and valuable of Mr. Cavendish's chemical experiments were published in two papers, entitled *Experiments on Air*; the first inserted in the Philosophical Transactions for 1784 (vol. lxxiv. p. 119); and the second in the Transactions for 1785 (vol. lxxv. p. 372). The object of these experiments was to determine what happened during the *phlogistication of air*, as it was at that time termed; that is, the change which air underwent when metals were calcined in contact with it, when sulphur or phosphorus was burnt in it, and in several similar processes. He showed, in the first place, that there was no reason for supposing that carbonic acid was formed, except when some animal or vegetable substance was present; that when hydrogen gas was burnt in contact with air or oxygen gas, it combined with that gas and formed water; that nitrous gas, by combining with with the oxygen of the atmosphere, formed nitrous acid; and that when oxygen and azotic gas are mixed in the requisite proportions, and electric sparks passed through the mixture, they combine and form nitric acid. The first of these opinions occasioned a controversy between Mr. Cavendish and Mr. Kirwan, who had maintained that carbonic acid is always produced when air is phlogisticated. Two papers on this subject are published in the Philosophical Transactions by Mr. Kirwan (Phil. Trans. 1784, vol. lxxiv. p. 154 and 178); and one by Mr. Cavendish (Ibid. p. 170); each remarkable examples of the peculiar manner of the respective writers. All the arguments of Kirwan are founded on the experiments of others: he displays great reading, and a strong memory; but does not discriminate between the merits of the chemists on whose authority he founds his opinions. Mr. Cavendish, on the other hand, never advances a single opinion which he has not put to the test of experiment; and never suffers himself to go farther than his experiments will warrant. Whatever is not accurately determined by unexceptionable trials, is merely stated as a conjecture, upon which little stress is laid.

In the first of these celebrated papers, Mr. Cavendish has drawn a comparison between the phlogistic and antiphlogistic theories; has shown that each of them is capable of explaining the phenomena of chemistry in a satisfactory manner; that it is impossible to demonstrate the truth of either; and he has given the reasons which induced him to prefer the phlogistic theory to the other, which the French chemists were unable to refute, and which they were wise enough not to notice. Nothing can be a more striking proof of the influence of fashion even in science, and of the unwarrantable precipitation with which opinions are rejected or embraced by philosophers, than the total inattention paid by the chemical world to this admirable dissertation. Had Mr. Kirwan adopted the opinions of Mr. Cavendish, when he undertook the defence of phlogiston, instead of trusting to the vague experiments of inaccurate chemists, he would never have been obliged to yield to his French antagonists, and the antiphlogistic theory would never have gained ground.

Such were the chemical papers published by Mr. Cavendish. They contain five notable discoveries; all of them brought nearly to perfection by that illustrious author. These are—1. The nature and properties of hydrogen gas. 2. The solvent of lime in water when the lime is deposited by boiling. 3. The exact proportion of the constituents of atmospherical air, and the fact that these constituents never sensibly vary. 4. The composition of water. 5. The composition position of nitric acid. It is proper to add, that Mr. Cavendish was the first person who showed that potash has a stronger affinity for acids than soda. His experiments on the subject are to be found in a paper on mineral waters, published in the Philosophical Transactions by Dr. Donald Monro.

II. *Electrical Papers.*—The papers published by Mr. Cavendish on electricity are only two; but they constitute, perhaps, the most elaborate of all his investigations. His first paper is entitled, *An Attempt to explain some of the principal Phenomena of Electricity by means of an Elastic Fluid.* (Phil. Trans. 1771, vol. lxi. p. 584.) This paper is very long; and contains a very complete mathematical theory of electricity, deduced from the hypothesis that there exists an electric fluid, the particles of which repel each other, but are attracted by all other matter with a force inversely as some less power of the distance than the cube. This paper is not susceptible of abridgement; but it deserves the careful study of every electrician. Æpinus, about the same time, adopted the same hypothesis, and published a book on the same subject, replete with valuable information; but he does not carry the subject quite so far as Mr. Cavendish. Besides the value of this paper in a philosophical point of view, it claims the attention of mathematicians on account of the neatness, simplicity, and shortness, of the demonstrations. The only other electrical paper of Mr. Cavendish consists in a set of experiments made on purpose to elucidate the shock communicated by the torpedo, and to show that it was consistent with the known properties of the electric fluid. (Phil. Trans. 1776, vol. lxvi. p. 196.) This paper is marked by the sagacity and the patient industry which distinguishes every thing that came from the hands of Mr. Cavendish. He succeeded perfectly in the object which he had in view; though the discovery of the galvanic pile has thrown additional light on the nature of the organ by which the torpedo produces that remarkable effect.

III. *Meteorological Papers.*—The meteorological papers avowedly written by Mr. Cavendish are only two; and, perhaps, in strict propriety, the term meteorological ought not to be applied to them. The first of these is an account of the meteorological instruments used at the Royal Society house. (Phil. Trans. 1776, vol. lxvi. p. 375.) These are the thermometer, barometer, rain-gage, wind-gage, hygrometer, variation compass, and dipping needle. Important observations and instructions are given respecting the proper mode of constructing and using the thermometer, the variation compass, and the dipping needle. Mr. Cavendish's second meteorological paper is a calculation of a remarkable luminous arch, seen Feb. 23, 1784. He shows that its height was not less than fifty-two statute miles, and that it could not exceed seventy-one statute miles. (Phil. Trans. 1790, vol. lxxx. p. 101.) There is strong reason to conclude, from the style, and from the train of observations contained in it, that a Report of a Committee of the Royal Society, appointed to consider the best method of adjusting the fixed points of thermometers, and of the precautions necessary to be used in making experiments with those instruments (Phil. Trans. 1777, vol. lxvii. p. 816), was written by Mr. Cavendish. But as this is not quite certain, we have not reckoned it among the number of his papers.

IV. *Astronomical Papers.*—The astronomical papers of Mr. Cavendish amount to three, and display the same sagacity and patient industry as his papers on the other departments of science. The first of these papers is on the civil year of the Hindoos, and its divisions; with an account of three Hindoo almanacs, belonging to Charles Wilkins, esq. (Phil. Trans. 1792, vol. lxxxii. p. 383.) The second paper is a letter to Mr. Mendoza y Rios, giving a new rule for finding the longitude by what is called the lunar observations. (Phil. Trans. 1797, vol. lxxxvii. p. 43.) Mr. Cavendish's last astronomical paper, and the last paper which he ever published, is an account of a set of experiments made to determine the density of the earth by rendering sensible the attraction of small quantities of matter. (Phil. Trans. 1798, vol. lxxxviii. p. 469.) The apparatus was originally contrived by Mr. John Mitchel; but was new modelled, and greatly improved, by Mr. Cavendish. The result of these experiments was, that the mean density of the earth does not differ from 5·48 by so much as $\frac{1}{14}$ of the whole; that is, that it is not less than 5·09, nor greater than 5·87. The experiments of Dr. Maskelyne on Schehallion, when corrected by the late observations of Mr. Playfair, give the density of the earth 4·867. These two sets of observations compared, would induce one to suspect that the mean density of the earth does not differ much from 5.—*Thomson's Annals of Philosophy.*

